# Echocardiographic and platelet markers of cardiovascular strain in sickle cell disease with superimposed preeclampsia: a perspective

**DOI:** 10.1097/MS9.0000000000004332

**Published:** 2025-11-17

**Authors:** Emmanuel Ifeanyi Obeagu

**Affiliations:** Department of Biomedical and Laboratory Science, Africa University, Mutare, Zimbabwe

**Keywords:** cardiovascular strain, echocardiography, platelet activation, preeclampsia, sickle cell disease

## Abstract

Sickle cell disease (SCD) and preeclampsia (PE) are both conditions associated with significant vascular dysfunction and increased cardiovascular risk, particularly during pregnancy. When PE is superimposed on SCD, the compounded pathophysiological effects exacerbate endothelial damage, inflammation, and hemodynamic stress, posing substantial risks to maternal and fetal health. Echocardiography offers a valuable non-invasive approach to assess cardiac function and detect subclinical cardiovascular alterations in pregnant women with SCD complicated by PE. Key echocardiographic markers, including left ventricular diastolic dysfunction, pulmonary hypertension, and right ventricular impairment, provide insight into the extent of cardiovascular compromise. Concurrently, platelet activation markers such as mean platelet volume and surface expression of adhesion molecules reflect a heightened pro-thrombotic state that contributes to vascular injury and disease progression. Integrating echocardiographic findings with platelet biomarkers may enhance risk stratification and guide targeted interventions to mitigate cardiovascular complications in this high-risk population. Further research is needed to establish standardized protocols and explore therapeutic avenues aimed at reducing maternal morbidity and improving pregnancy outcomes in women with SCD and superimposed PE.

## Introduction

SCD is a hereditary hemoglobinopathy characterized by the production of abnormal hemoglobin S, which causes red blood cells to deform into a sickle shape under hypoxic conditions^[1,2]^. This abnormality leads to recurrent vaso-occlusive episodes, chronic hemolysis, and widespread endothelial dysfunction. SCD is associated with multi-organ complications, including cardiopulmonary involvement, which remains a leading cause of morbidity and mortality. Pregnancy in women with SCD is recognized as high risk due to increased maternal and fetal complications, driven largely by the complex pathophysiology of the disease^[3-6]^. Preeclampsia (PE), a hypertensive disorder occurring after 20 weeks of gestation, is characterized by new-onset hypertension and organ dysfunction, including renal impairment and proteinuria. It is a major cause of maternal and perinatal morbidity worldwide. The pathogenesis of PE involves abnormal placental development, systemic endothelial activation, oxidative stress, and inflammation, resulting in widespread vascular dysfunction. When superimposed on pre-existing conditions such as SCD, PE markedly increases cardiovascular strain and worsens pregnancy outcomes^[7-11]^. The coexistence of SCD and superimposed PE creates a synergistic pathophysiological burden on the cardiovascular system. Both disorders promote endothelial injury, heightened inflammatory responses, and a pro-thrombotic state that exacerbates vascular damage. This results in increased hemodynamic load, ventricular remodeling, and potential heart failure^[11]^.

Echocardiography is a cornerstone diagnostic tool in assessing cardiac structure and function in various clinical settings. In patients with SCD, echocardiographic evaluation frequently reveals left ventricular hypertrophy, diastolic dysfunction, and elevated pulmonary artery pressures indicative of pulmonary hypertension. The addition of PE can worsen these findings by further impairing cardiac function and increasing afterload. Advanced echocardiographic techniques such as tissue Doppler imaging and myocardial strain analysis provide sensitive detection of subclinical myocardial impairment, allowing for timely intervention^[12–14]^. Platelet activation is another critical component in the pathophysiology of both SCD and PE. Chronic hemolysis and endothelial damage in SCD lead to persistent platelet activation, contributing to vaso-occlusion and thrombosis. Similarly, PE is characterized by increased platelet aggregation and consumption, contributing to a hypercoagulable state. Biomarkers such as mean platelet volume (MPV), platelet-to-lymphocyte ratio (PLR), and surface markers like P-selectin reflect platelet reactivity and have been associated with disease severity and cardiovascular risk in these conditions^[15]^. The integration of echocardiographic parameters with platelet activation markers presents a promising approach for comprehensive cardiovascular risk assessment in pregnant women with SCD and superimposed PE. Such combined assessment may improve early detection of cardiovascular strain, guide therapeutic decisions, and inform prognostication. However, data on the interaction between these markers in this population remain limited, underscoring the need for further research^[16,17]^. This review aims to synthesize current evidence on the use of echocardiographic and platelet markers in detecting cardiovascular strain in SCD complicated by superimposed PE.

## Aim

This review aims to comprehensively examine the role of echocardiographic and platelet activation markers in detecting and assessing cardiovascular strain in pregnant women with sickle cell disease complicated by superimposed PE.

## Pathophysiological overlap between sickle cell disease and PE

SCD and PE are distinct clinical entities with complex pathophysiologies, yet they share remarkable overlaps that significantly impact maternal cardiovascular health when coexisting. At the core of both conditions lies endothelial dysfunction, a critical factor driving vascular instability and organ damage^[17–19]^. In SCD, recurrent hemolysis and vaso-occlusion initiate a cascade of oxidative stress and inflammation, damaging the vascular endothelium and impairing its ability to regulate vasodilation, coagulation, and barrier function. Similarly, PE stems from abnormal placentation and inadequate remodeling of maternal spiral arteries, resulting in placental ischemia that triggers systemic endothelial activation and widespread inflammatory responses^[20–23]^. This shared endothelial injury leads to the release of numerous vasoactive substances, including endothelin-1 and reduced bioavailability of nitric oxide, tipping the balance toward vasoconstriction and hypertension. In both SCD and PE, the disruption of this delicate equilibrium fosters increased vascular permeability and heightened sensitivity to vasoconstrictive stimuli, contributing to the development of hypertension and end-organ damage. The compounded vascular insult in pregnancies complicated by both SCD and superimposed PE amplifies these processes, increasing the risk of adverse cardiovascular events^[24,25]^. Beyond endothelial dysfunction, a hypercoagulable state characterizes both diseases. SCD is marked by chronic platelet activation and enhanced thrombin generation, driven by hemolysis-induced exposure of phosphatidylserine and circulating microparticles. PE similarly involves platelet activation and consumption due to systemic inflammation and endothelial disruption. This pro-thrombotic environment exacerbates vascular occlusion and tissue ischemia, particularly in the microcirculation, further straining the cardiovascular system. Consequently, pregnant women facing the dual burden of SCD and PE experience compounded hemodynamic challenges, including increased cardiac output demands, pulmonary hypertension, and ventricular remodeling, all of which contribute to cardiovascular strain^[26–28]^.

## Echocardiographic markers of cardiovascular strain

Echocardiography is an indispensable, non-invasive imaging modality widely used to assess cardiac structure and function, especially in high-risk populations such as pregnant women with SCD complicated by superimposed PE. This tool provides real-time insights into myocardial performance, hemodynamic burden, and pulmonary vascular pressures, all of which are crucial for detecting and monitoring cardiovascular strain^[29]^. In patients with SCD, echocardiographic abnormalities are common due to chronic anemia-induced high cardiac output states and progressive vascular damage. Left ventricular hypertrophy (LVH) is frequently observed, reflecting the heart’s compensatory response to increased workload. Diastolic dysfunction is another hallmark, indicating impaired myocardial relaxation and increased filling pressures that can precede overt heart failure. When PE is superimposed, these echocardiographic changes often worsen, as hypertensive stress and endothelial dysfunction further compromise cardiac function^[30,31]^.

Pulmonary hypertension (PH) is a significant concern in SCD and can be effectively screened using echocardiography by estimating pulmonary artery systolic pressure (PASP) through tricuspid regurgitant jet velocity. Elevated PASP correlates with increased morbidity and mortality and may be exacerbated by PE due to heightened systemic vascular resistance and placental insufficiency. Right ventricular (RV) function assessment, including RV size and fractional area change, is essential, as RV overload may result from PH and contribute to cardiovascular decompensation^[12,32]^. Advanced echocardiographic techniques, such as tissue Doppler imaging (TDI) and speckle-tracking echocardiography, offer enhanced sensitivity in detecting subclinical myocardial dysfunction. Parameters like E/E’ ratio obtained via TDI can estimate left ventricular filling pressures, providing early clues to diastolic dysfunction. Myocardial strain imaging evaluates the deformation of cardiac muscle during contraction and relaxation, uncovering subtle impairments before changes in ejection fraction become apparent. These modalities are particularly valuable in pregnant women with SCD and PE, where early intervention can prevent progression to overt cardiac failure (Table 1) ^[33]^.

**Table 1:** Key echocardiographic markers of cardiovascular strain in SCD with superimposed preeclampsia

Echocardiographic Parameter	Clinical Significance	Interpretation in SCD + Preeclampsia
Left Ventricular Diastolic Function (E/A ratio, E/e’ ratio)	Early indicator of myocardial stress and impaired relaxation	Often shows elevated filling pressures due to combined anemia-related volume load and increased afterload from preeclampsia
Global Longitudinal Strain (GLS)	Detects subclinical systolic dysfunction	Reduced strain may indicate early left ventricular impairment before overt heart failure develops
Right Ventricular Systolic Pressure (RVSP)	Surrogate marker for pulmonary hypertension	Elevated RVSP reflects pulmonary vascular stress, common in SCD with superimposed preeclampsia
Left Ventricular Mass Index (LVMI)	Measures hypertrophic remodeling	Increased LVMI reflects chronic volume overload and adaptive hypertrophy
Cardiac Output & Stroke Volume	Hemodynamic assessment	May be altered due to high-output state in anemia combined with systemic vasoconstriction from preeclampsia

## Platelet activation and hemostatic markers

Platelet activation plays a pivotal role in the pathophysiology of both SCD and PE, contributing to vascular dysfunction and heightened cardiovascular strain. In SCD, chronic hemolysis and ongoing endothelial injury create a pro-inflammatory and pro-thrombotic milieu that perpetuates platelet hyperreactivity. This hyperactivation promotes vaso-occlusion, microvascular ischemia, and exacerbation of end-organ damage. Similarly, PE is characterized by systemic endothelial activation and an imbalance between pro- and anti-coagulant factors, leading to platelet aggregation and consumption that further amplify vascular injury^[15,34,35]^. Several platelet-related biomarkers have been investigated to assess disease activity and cardiovascular risk in these conditions. MPV, a readily available laboratory parameter, reflects platelet size and activity; elevated MPV values are frequently observed in both SCD and PE, indicating increased platelet reactivity. The PLR, an emerging inflammatory marker, has also demonstrated correlation with disease severity and adverse outcomes, reflecting the intricate interplay between thrombosis and inflammation^[16,36,37]^. Beyond routine hematological indices, molecular markers of platelet activation provide deeper insight into the underlying hemostatic disturbance. Surface expression of P-selectin (CD62P) and CD40 ligand (CD40L) on platelets mediates adhesion to the endothelium and leukocytes, fostering inflammation and thrombus formation. Elevated plasma levels of thromboxane A2 and β-thromboglobulin are indicative of enhanced platelet degranulation and activation. The presence of circulating platelet-derived microparticles further propagates coagulation cascades and endothelial dysfunction (Table 2) ^[38,39]^.

**Table 2:** Platelet markers reflecting vascular and cardiovascular stress in SCD with superimposed preeclampsia

Platelet Marker	Mechanistic Insight	Clinical Relevance
Mean Platelet Volume (MPV)	Indicates platelet reactivity and size	Elevated MPV correlates with heightened thrombotic potential and cardiovascular risk
Platelet Distribution Width (PDW)	Reflects heterogeneity in platelet size	Increased PDW may signal ongoing platelet activation and turnover
Plateletcrit (PCT)	Total platelet mass	Elevated PCT may indicate increased thrombotic burden in systemic microvascular stress
Platelet Microparticles (PMPs)	Procoagulant vesicles from activated platelets	High PMP levels indicate endothelial injury, oxidative stress, and a hypercoagulable state
Platelet Count	Quantitative marker	May be reduced due to consumption or sequestration in microvasculature, highlighting systemic stress

## Clinical implications

The coexistence of SCD and superimposed PE presents a formidable challenge in obstetric care due to the compounded cardiovascular strain that significantly elevates maternal and fetal morbidity and mortality risks. Early and accurate assessment of cardiovascular status through echocardiographic and platelet activation markers offers a valuable approach to risk stratification and clinical management in this vulnerable population^[40]^. Echocardiography facilitates the identification of subclinical cardiac dysfunction before the onset of overt symptoms, allowing for timely interventions such as optimized blood pressure control, volume management, and consideration of advanced therapies to reduce cardiac workload. Recognition of elevated pulmonary artery pressures and right ventricular dysfunction prompts vigilance for pulmonary hypertension—a major contributor to adverse outcomes in SCD pregnancies—and guides appropriate monitoring and referral to specialized care^[41]^.

Similarly, the assessment of platelet activation and hemostatic markers provides insight into the thrombotic risk inherent in these patients. Elevated platelet reactivity may justify the cautious use of antiplatelet or anticoagulant therapies, tailored to balance maternal benefits against bleeding risks. Serial monitoring of platelet indices can also aid in evaluating treatment response and disease progression^[35,42]^. Integrating echocardiographic findings with platelet biomarker profiles enables a comprehensive cardiovascular evaluation, which may improve clinical decision-making and individualized care plans. Moreover, these markers can serve as endpoints in clinical trials exploring novel therapeutic strategies, including the role of antiplatelet agents, vasodilators, or antioxidant therapies in mitigating cardiovascular complications^[43]^. In resource-limited settings, where access to advanced imaging may be constrained, platelet markers could serve as accessible surrogates for cardiovascular strain, facilitating early referral and intervention. Ultimately, multidisciplinary collaboration involving obstetricians, hematologists, cardiologists, and maternal-fetal medicine specialists is essential to optimize outcomes for pregnant women with SCD complicated by PE^[44,45]^. Future research should focus on establishing standardized echocardiographic protocols and validating platelet biomarkers in larger cohorts, to refine predictive models and develop evidence-based guidelines tailored to this high-risk group.

## Conclusion

Sickle cell disease complicated by superimposed PE imposes a significant burden of cardiovascular strain, driven by intertwined mechanisms of endothelial dysfunction, inflammation, and thrombosis. Echocardiographic evaluation offers a vital window into cardiac structural and functional changes, enabling early detection of subclinical myocardial impairment and pulmonary hypertension. Concurrently, platelet activation markers provide critical insights into the pro-thrombotic state that exacerbates vascular injury and contributes to adverse outcomes. The combined use of echocardiographic parameters and platelet biomarkers holds promise for enhancing cardiovascular risk stratification and guiding individualized management strategies in this vulnerable population. Such integrated monitoring may facilitate timely interventions, reduce maternal and fetal morbidity, and improve overall pregnancy outcomes.
